# Understanding the Impact of ErbB Activating Events and Signal Transduction on Antigen Processing and Presentation: MHC Expression as a Model

**DOI:** 10.3389/fphar.2016.00327

**Published:** 2016-09-26

**Authors:** Anna E. Kersh, Maiko Sasaki, Lee A. Cooper, Haydn T. Kissick, Brian P. Pollack

**Affiliations:** ^1^Medical Scientist Training Program, Emory University School of MedicineAtlanta, GA, USA; ^2^Atlanta VA Medical CenterDecatur, GA, USA; ^3^Department of Biomedical Informatics, Emory University School of MedicineAtlanta, GA, USA; ^4^Department of Biomedical Engineering, Georgia Institute of TechnologyAtlanta, GA, USA; ^5^Department of Urology, Emory University School of MedicineAtlanta, GA, USA; ^6^Department of Dermatology, Emory University School of MedicineAtlanta, GA, USA

**Keywords:** MHC class I, MHC class II, EGFR, ErbB receptors, immunology, signal transduction, gene expression regulation

## Abstract

Advances in molecular pathology have changed the landscape of oncology. The ability to interrogate tissue samples for oncogene amplification, driver mutations, and other molecular alterations provides clinicians with an enormous level of detail about their patient’s cancer. In some cases, this information informs treatment decisions, especially those related to targeted anti-cancer therapies. However, in terms of immune-based therapies, it is less clear how to use such information. Likewise, despite studies demonstrating the pivotal role of neoantigens in predicting responsiveness to immune checkpoint blockade, it is not known if the expression of neoantigens impacts the response to targeted therapies despite a growing recognition of their diverse effects on immunity. To realize the promise of ‘personalized medicine’, it will be important to develop a more integrated understanding of the relationships between oncogenic events and processes governing anti-tumor immunity. One area of investigation to explore such relationships centers on defining how ErbB/HER activation and signal transduction influences antigen processing and presentation.

## Introduction and Overview

Stated simply, oncogenic events are not immunologically null (Seliger, 2014). Defining the immunologic impact of oncogenic events will be a critical area of research as advances in molecular diagnostics parallel the expansion and availability of cancer immunotherapy (Whiteside et al., 2016). Alterations in DNA sequence, copy number variation (CNV), transcriptional profile, and epigenetics can all influence the expression of genes of the immune system, immunologic processes, and ultimately anti-tumor immunity. The importance of antigen processing and presentation in this regard is underscored by recent studies that have defined the impact of mutation-derived neoantigens on the response to immune checkpoint blockade (Sorensen et al., 2009; van Rooij et al., 2013; Schumacher et al., 2014; Snyder et al., 2014; Kelderman et al., 2015; Le et al., 2015; Van Allen et al., 2015). These recent findings, further highlight the importance of defining interactions between oncogenes, oncogenic signal transduction, and the antigen processing machinery (APM) (Seliger, 2014). Moreover, because the APM plays a critical role in cancer immunoediting, understanding how anti-cancer therapies (of all types) influence the APM will be equally important (Galluzzi et al., 2012; Wargo et al., 2014; Okwan-Duodu et al., 2015; Ward et al., 2016). The goal of this article is to provide a framework for understanding interactions between oncogenic ErbB/HER signal transduction and the APM, using the expression of major histocompatibility complex (MHC) molecules as a model.

### Oncogenes and the APM – General Considerations

**FIGURE 1:**
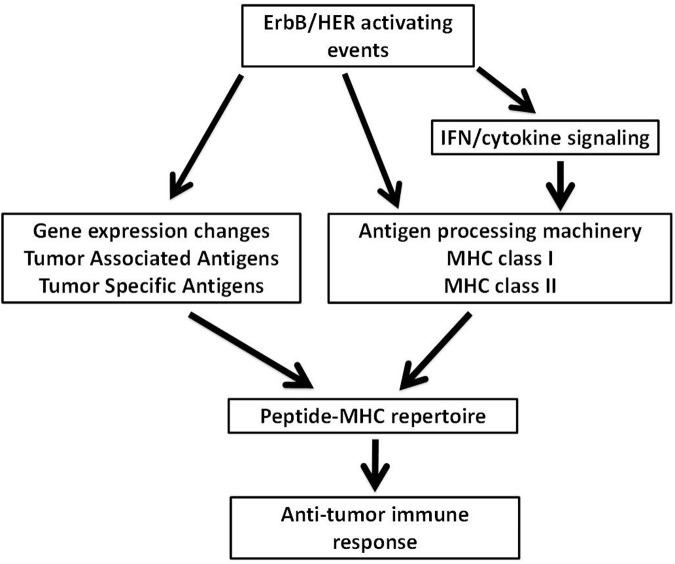
**Possible mechanisms through which ErbB/HER oncogenic activating events influence the peptide-MHC (pMHC) repertoire.** ErbB/HER oncogenic events may influence the peptide-MHC repertoire via several possible mechanisms. ErbB/HER oncogenic activation alters the transcriptome of tumor cells which may include the expression of tumor associated antigens and tumor specific antigens (TSA) such as neoantigens. By altering the transcriptome of tumor cells, ErbB/HER oncogenic events influence the pool of available proteins available for antigen processing and presentation. In addition, ErbB/HER oncogenic signaling may influence the expression of components of the antigen processing machinery (APM) including MHCI and MHCII molecules directly or indirectly by altering the cellular response to cytokines. This will alter the nature and density of the pMHC repertoire. Qualitatively and quantitatively, the nature of the pMHC repertoire may influence T cell-mediated anti-tumor immune responses.

Recent studies confirming the relevance of neoantigens, also referred to as tumor specific antigens (TSA), provides enormous rationale to understand how oncogenic events impact the ability of tumor cells to process and present antigenic peptides. Moreover, being able to pharmacologically manipulate the expression of proteins that make up the APM takes on additional relevance since defects within the APM are common in human cancer and such alterations will likely influence the peptide-MHC (pMHC) repertoires presented by tumors cells (Seliger et al., 2000; Garrido et al., 2016). The dual ability of ErbB/HER signaling to modulate large transcriptional programs and influence MHC expression suggests that alterations in the ErbB/HER family of ligands and/or receptors will change the pMHC repertoire present at the surface of tumor cells. Alterations of ErbB/HER receptors and ligands occurring in human cancer are shown in **Tables 1** and **2**. This concept was supported by the fact that ErbB/HER ligands and receptors can greatly influence the cellular transcriptome, which in turn influences the pMHC repertoire (Choi et al., 2007; Fortier et al., 2008; Nagashima et al., 2008). In addition, the complex nature of ErbB/HER signaling that results from having numerous ligands, receptors, and mechanistically distinct activating events, supports the idea that there will be differential effects on MHC expression. While it will likely be cell-context dependent, defining ErbB/HER oncogenic signaling in this manner provides an experimental approach that can further our understanding of interactions between oncogenes and immunity. Moreover, this approach lends itself to validation using cell-based systems, genetically engineered mouse models, and human pathology samples. Some of these concepts are shown in **Figure 1**.

**Table 1:** Alterations of ErbB/HER receptors in human cancer.

Receptor	Mutation/Modification	Cancer	Reference
EGFR/ErbB1/HER1	Overexpression	Colorectal carcinoma	Moroni et al., 2005
	Substitutions G719S, L858R	NSCLC	Paez et al., 2004
	Activating in-frame kinase domain deletions	NSCLC	Lynch et al., 2004
	Overexpression, activating deletions (truncation), EGFRvIII (801 bp in-frame deletion of extracellular domain, activating)	Glioblastoma	Watanabe et al., 1996; Frederick et al., 2000; Paff et al., 2014
	Overexpression and cytoplasmic expression	Cutaneous SCC	Canueto et al., 2016
	Overexpression	Head and neck SCC	Grandis and Tweardy, 1993
	Overexpression	Esophageal adenocarcinoma	Al-Kasspooles et al., 1993
ErbB2/HER2/NEU	Overexpression	Breast carcinoma	Carey et al., 2006; Howlader et al., 2014
	Overexpression	Gastric carcinoma	Falck and Gullick, 1989; Tanner et al., 2005; Yano et al., 2006; Hofmann et al., 2008
	Insertion, overexpression	NSCLC	Weiner et al., 1990; Oxnard et al., 2013
	Overexpression	Bladder carcinoma	Neal et al., 1985; Sato et al., 1992; Sauter et al., 1993
	Overexpression	Pancreatic carcinoma	Yamanaka et al., 1993b; Lei et al., 1995
	Overexpression	Ovarian carcinoma	Meden and Kuhn, 1997
	Overexpression	Endometrial carcinoma	Saffari et al., 1995; Rolitsky et al., 1999
	Overexpression	Head and neck SCC	Beckhardt et al., 1995
	Overexpression	Esophageal adenocarcinoma	Al-Kasspooles et al., 1993
ErbB3/HER3	Substitution V855A (homologous to EGFR-L858R)	NSCLC	Umelo et al., 2016
	Overexpression	Oral SCC	Shintani et al., 1995
ErbB4/HER4	Missense mutations resulting in increased kinase activity	Melanoma	Prickett et al., 2009
	Missense/insertions/deletions in kinase domain	Gastric, breast, lung and colorectal carcinomas	Soung et al., 2006

**Table 2:** Alterations of ErbB/HER ligands in human cancer.

Ligand	Mutation/Modification	Cancer	Reference
Epidermal growth factor (EGF)	Overexpression	Pancreatic	Yamanaka et al., 1993a
	Point mutation	Gastric	Wu et al., 2015
Transforming growth factor-α (TGF-α)	Overexpression	Prostate	Scher et al., 1995
	Overexpression	Pancreatic	Yamanaka et al., 1993a
	Overexpression	Lung, ovarian, colon	Salomon et al., 1995; Rusch et al., 1997
Neuregulin/ Heregulin 1 (Nrg1/Hrg1)	Overexpression	Breast carcinoma	Raj et al., 2001; Slattery et al., 2013
	Chromosomal translocation	Breast carcinoma	Huang et al., 2004
Amphiregulin (AREG)	Overexpression	Ovarian	Niikura et al., 1997

### A Brief Overview of MHC Molecules

Major histocompatibility complex molecules are cell surface glycoproteins that function to present self- and antigen-derived peptides to cells of the immune system. In humans, MHC molecules are encoded by human leukocyte antigen (HLA) genes and these terms are often used interchangeably. MHC class I (MHCI) molecules consist of two polypeptide chains, the alpha chain (HLA-A, B, C) and ß2-microglobulin which are non-covalently associated on the cell surface. The alpha-1 and alpha-2 domains of the MHC class I molecule form the peptide binding cleft which binds and presents cytosol-derived peptides that are 8-11 amino acids in length to CD8 T lymphocytes (Comber and Philip, 2014). As MHCI molecules function in concert with CD8 T cells for immune surveillance against infections and malignancies, these molecules are expressed on the surface of virtually all nucleated cells and MHCI expression can be further increased (induced) by type I and type II interferons (IFNs) as well as other cytokines. Underscoring their important role in controlling malignancies and infections is the fact that tumor cells and microbes alike such as the human immunodeficiency virus (HIV) and *Mycobacterium tuberculosis* (Mtb) harbor mechanisms that down-regulate MHCI expression to facilitate immune escape (Ferris et al., 2006; Pennini et al., 2006; Choma et al., 2015; Concha-Benavente et al., 2016).

MHC class II (MHCII) molecules function to present antigen to CD4 T lymphocytes generating helper T cell responses that are critical for effective adaptive immune responses against infection and cancer (DeSandro et al., 1999; Accolla et al., 2014). Each MHCII molecule (HLA-DR, DP, DQ) is composed of an alpha and beta polypeptide chain that non-covalently associate at the cell surface with one subunit from each chain forming the peptide binding cleft. MHCII molecules bind peptides of 13-17 amino acids in length that are generated by proteolysis in lysosomes and endosomes and are constitutively expressed on the surface of antigen presenting cells (APCs) such as B cells, macrophages, and dendritic cells (DCs; Roche and Furuta, 2015).

In order to understand how oncogenic signal transduction might influence the expression of MHC molecules, it is important to review some aspects of MHC expression regulation. In general, though not exclusively, MHC molecules are regulated transcriptionally and epigenetically (van den Elsen et al., 2004; Choi et al., 2011; Kobayashi and van den Elsen, 2012). This regulation is orchestrated at several levels involving complex interactions between regulatory DNA sequences, within the MHC locus (such as promoters and enhancers), DNA-binding transcription factors (TFs) that bind these sequences, transcriptional co-activators (NLRC5/CITA for MHCI and CIITA for MHCII) and the formation of complex looping structures that involve interactions with epigenetic enzymes and chromatin (Gobin et al., 2001; Meissner et al., 2012). There are many excellent detailed reviews available on the regulation of MHCI and MHCII molecules (van den Elsen et al., 1998; van den Elsen, 2011; Devaiah and Singer, 2013; Neerincx et al., 2013).

When considering interactions between oncogenic signaling and MHCI expression, two types of expression need to be considered. Constitutive expression refers to the level of MHCI molecules expressed under physiologic conditions and varies between different tissues due in part to differences in epigenetic marks (Kotekar et al., 2008). In addition to constitutive expression, increases that occur in response to cytokines are referred to as inducible expression. Defects in both types of MHCI expression occur in human cancer (Garrido et al., 2010). Mechanistically, constitutive MHCI expression is regulated by distinct regions within MHCI promoters that are binding sites for TFs such as NFkB, IRF-1, and CREB. The inducible expression of MHCI molecules occurs in response to cytokines such as type I and type II interferons (IFNs) and tumor necrosis factor-alpha (TNF-α) and is mediated through changes in TFs, co-activators, and other proteins that occur in response to the inducing cytokine (van den Elsen, 2011). Thus, when considering interactions between ErbB/HER signaling and MHC expression, the status of both constitutive and inducible MHCI expression warrant attention.

In contrast to MHCI, MHCII is expressed constitutively only on specialized cells of the immune system such as DCs and B cells. The expression of MHCII is regulated largely via the activity of the MHCII co-activator (CIITA) which itself is regulated by distinct promoters that are active in DCs and B cells (named pI and pIII, respectively). Interestingly, aberrant constitutive MHCII expression occurs on some tumor cells such as melanoma (Martins et al., 2007; Degenhardt et al., 2010). Despite this restricted constitutive expression pattern, MHCII molecules are inducible by IFN-γ in most cell types; a unique feature of IFN-γ. The fact that MHCI and MHCII molecules are inducible by cytokines is highly relevant to ErbB/HER signaling because as outlined in more detail below, there are functional links between cytokine signaling and ErbB/HER signaling. Moreover, ErbB/HER signaling can also influence the expression of the TFs that bind to the promoters of HLA class I genes such as NFκB and IRF-1 (Andersen et al., 2008; Shostak and Chariot, 2015). Thus, ErbB/HER signaling pathways are well poised to alter MHC expression (positively or negatively) via mechanisms that are not entirely understood.

### MHC Molecules and Cancer

In the setting of cancer, MHC molecules play the pivotal role of presenting processed tumor antigens to CD4 and CD8 lymphocytes in order to generate a tumor-specific cytotoxic response (Seliger, 2008b; Hastings, 2013). As tumor antigens are ultimately derived from self, barriers to the activation of an antitumor T cell response exist intrinsically as T cells with affinity for self-antigens are deleted during T cell development. Further, tumor cells can create an immunosuppressive microenvironment to suppress anti-tumor T cell responses (Kim et al., 2006). As mentioned earlier, one mechanism through which tumor cells evade detection by the immune system is the down regulation of the surface expression of MHCI molecules. This can occur via many mechanisms including the genetic loss of the MHC locus, epigenetic silencing, and many others (Marincola et al., 2000; Seliger, 2008a,b, 2014; van der Burg et al., 2016). Importantly, while some of these defects are irreversible, others are not and in these cases the expression of MHCI molecules can be corrected or ‘rescued’ by cytokines (such as IFNs) and medications such as metformin, inhibitors of DNA methylation, histone deacetylase (HDAC) inhibitors, and other approaches including targeted therapies as outlined later in this review (Seliger and Pfizenmaier, 1989; Komatsu and Hayashi, 1998; Magner et al., 2000; Lopez-Albaitero et al., 2006; Khan et al., 2008; Han et al., 2011; Lampen and van Hall, 2011; Oliveras-Ferraros et al., 2012). Many immune-based therapies hinge on the generation of effector CD4 and/or CD8 T cells. As such, being able to increase MHCI and/or MHCII levels pharmacologically on tumor cells would be an attractive complement to CD4/CD8-based immunotherapy because CD4- and CD8 T cell responses are functionally influenced by the pMHC density present at the cell surface. For MHCI, as pMHCI density is increased, T cell activation becomes more efficient and the inhibition seen in the setting of an excessively long TCR-pMHCI interaction half-life is attenuated (Gonzalez et al., 2005). For MHCII, higher pMHCII density can elicit a more robust CD4 T cell response than the same pMHCII at a lower density (Vanguri et al., 2013). While it is less clear if increases in pMHC density can functionally alter an anti-tumor immune response, increasing pMHC density via the aforementioned approaches offers another possible avenue to manipulate interactions between tumor cells and therapeutic T cells.

In addition to their level of expression, the peptides bound to MHC molecules play a crucial role in anti-tumor immunity. As mentioned previously, the presentation of strongly antigenic peptides (such as neoantigens derived from mutations) via MHCI or MHCII can have an enormous impact on clinical responses to immunotherapies such as those targeting immune checkpoints (mediated by CTLA-4 signaling and the PD-1/PD-L1 axis) (Lu and Robbins, 2016; Ward et al., 2016). With this in mind, it will be crucial to understand how oncogenic events and anti-cancer therapies influence antigen processing, antigen presentation, and the pool of antigenic peptides that can be displayed on the cell surface of tumors via MHC molecules and detected by anti-tumor CD4- and/or CD8 T lymphocytes (Chang and Ferrone, 2007; Hastings, 2013).

## Linking ErbB/HER to the MHC

Given their canonical roles in cancer biology and immunology, respectively, it is not surprising that attempts to identify interactions between the ErbB/HER and MHC molecules goes back three decades (Schreiber et al., 1984). These initial papers examined the ability of EGFR ligands to influence MHC expression. For example, it was found that epidermal growth factor (EGF) treatment could reduce the binding of KE-2 antibodies (which recognize an epitope on the heavy chain of HLA class I molecules) to A431 cells; a finding that pre-dates the seminal paper by Sporn and Roberts (1985) regarding the autocrine secretion of growth factors such as TGF-α by cancer cells (Schreiber et al., 1984; Sporn and Roberts, 1985). In regards to MHCII, links between MHCII expression and ErbB/HER ligands dates back over two decades where it was demonstrated that the induction of HLA-DR molecules by IFN could be attenuated by ErbB/HER ligands EGF and/or transforming growth factor-α (TGFA) in thyroid epithelial cells and keratinocytes (Lahat et al., 1992; Mitra and Nickoloff, 1992). These early studies provided evidence that there were signals initiated by EGFR activation that had a repressive effect on the expression of MHCI and MHCII molecules at least in some cellular contexts.

When considering ErbB/HER-MHC interactions, it is important to consider the crosstalk that exists between ErbB/HER receptors and members of the cytokine receptor superfamily (Kaczmarski and Mufti, 1991; Prenzel et al., 2000). For example, in response to cytokines such as IFN-γ that induce MHC expression, ErbB/HER activation can occur via the protease-dependent release of ErbB/HER ligands (Burova et al., 2007). As a result, the cellular response to cytokines that induce MHC molecules involves the activation of ErbB/HER receptors. This places the EGFR and other members of the ErbB/HER family, as well as those pathways downstream, in a unique position to influence the cellular response to cytokines and thus MHC induction. This becomes even more important when one considers the dual nature of MHCI regulation described earlier since malignant cells can harbor defects in constitutive MHCI expression yet retain their responsiveness to cytokines. In such a scenario, it is possible that oncogenic ErbB/MHC signaling would be particularly impactful since MHCI expression on tumor cells would be effectively dependent on cytokines present in the tumor microenvironment for MHCI expression yet rendered less responsive to cytokines due to aberrant ErbB/HER signaling.

### ErbB/HER Ligands and MHC Expression

The ligands for ErbB/HER family members are EGF-family growth factors that include EGF, TGFA, amphiregulin (AREG), heparin-binding EGF-like growth factor (HBEGF), betacellulin (BTC), epigen (EPGN), epiregulin (EREG), and four neuregulins (NRG1-4; are also called heregulins) (Normanno et al., 2003a; Schneider and Wolf, 2009). These ligands bind ErbB/HER receptors with varying specificities and induce the formation of homo- and heterodimers creating a large number of possible receptor-ligand combinations (**Table 3**) (Roskoski, 2014). Through these interactions, ErbB/HER ligands initiate signal transduction via complex signaling networks that lead to large-scale changes in gene expression (Normanno et al., 2006). ErbB/HER ligands are widely expressed in epithelial tissues including those that give rise to the most common forms of human cancer such as the skin, gut, lungs and the aerodigestive tract. These ligands exert distinct effects based upon the receptors they engage and the nature of the downstream signals they transmit (O-Charoenrat et al., 2002; Zaiss et al., 2015). For example, by activating different receptors, ligands induce signals with distinct intensities and durations that in turn contribute to ligand-specific effects on gene expression (Nagashima et al., 2007). Moreover, ligands activating the same ErbB/HER receptor bind with different affinities causing distinct biological outcomes (Macdonald-Obermann and Pike, 2014). Transgenic and knockout mouse models have demonstrated these differences underscoring the fact that these ligands exert distinct effects even within the same tissue context (**Table 4**) (Normanno et al., 2006). In the skin, forced expression of TGF-α causes papillomas at sites of mechanical irritation, forced expression of AREG induces psoriatic-like lesions, and forced expression of betacellulin causes altered hair development (Vassar and Fuchs, 1991; Dominey et al., 1993; Cook et al., 1997; Schneider et al., 2008). Knockout studies have also illustrated the different roles for ErbB/HER ligands within the same tissue as mice deficient in both EGF and TGF-α expression show impaired prostate development while mice deficient only in TGF-α show increased proliferation of prostate tissue compared to wild-type (Abbott et al., 2003). This suggested that intact TGF-α signaling is required to control EGF-stimulated proliferation of prostate tissue. Similar observations were made in the central and peripheral nervous systems where EGF, TGF-α, and HBEGF are all expressed. Experiments in mice deficient in TGF-α demonstrated normal development and functioning of the peripheral nervous system suggesting EGF and HBEGF signaling are sufficient and compensate for a lack of TGF-α deficient signaling (Xian and Zhou, 2004). Despite the above phenotypes, the impact of ErbB/HER ligands on adaptive immune responses in these models are incompletely understood in part because these ligands were initially characterized for their ability to influence processes such as cell growth and survival. However, recently there has been a growing appreciation for the immune functions of ErbB/HER ligands.

**Table 3:** ErbB/HER receptors and their cognate ligands.

Ligands	ERBB Family Members			
	EGFR/ ERBB1/ HER1	ERBB2/ HER2/ NEU	ERBB3/ HER3	ERBB4/ HER4
EGF	+	No known ligands		
Epigen (EPGN)	+			
Transforming growth factor-α (TGF-α)	+			
Amphiregulin (AREG)	+			
Betacellulin (BTC)	+			+
Heparin-binding epidermal growth	+			+
factor-like growth factor (HB-EGF)
Epiregulin (EREG)	+			+
Neuregulin/Heregulin 1 (Nrg1/Hrg1)			+	+
Neuregulin/Heregulin 2 (Nrg2/Hrg2)			+	+
Neuregulin/Heregulin 3 (Nrg3/Hrg3)				+
Neuregulin/Heregulin 4 (Nrg4/Hrg4)				+

**Table 4:** Impact of ErbB/HER ligand knockout and overexpression.

Ligand(s)	Knockout (KO) Or overexpression (OE)	Tissues impacted and/or Phenotype	Reference
TGF-α	KO	Eyes, hair, and skin	Luetteke et al., 1999; Wong, 2003
AREG	KO	Mammary glands	Luetteke et al., 1999
EGF, TGF-α, AREG	KO	Eyes, hair, skin, mammary	Luetteke et al., 1999
EGF	OE	Infertile, growth retardation, shortened small intestine	Erwin et al., 1999; Chan and Wong, 2000; Wong et al., 2000
AREG	OE	Psoriasis-like dermatitis	Cook et al., 1997
TGF-α	OE	Epithelial hyperplasia, pancreatic metaplasia, breast carcinoma	Jhappan et al., 1990; Sandgren et al., 1990

One important role ErbB/HER ligands in the context of adaptive immunity was recently uncovered when it was demonstrated that regulatory T cells (T regs) express the EGFR and respond to EGFR ligands such as amphiregulin (AREG) (Zaiss et al., 2013). In addition, a role for AREG in ultraviolet radiation (UVR)-mediated immunosuppression has recently been reported (Meulenbroeks et al., 2015). The ability of immune cells including CD4 and CD8 T cells to produce AREG further underscores the expanding appreciation for the role of ErbB/HER ligands in adaptive immune responses including those against tumors (Zaiss et al., 2006; Kwong et al., 2010; Qi et al., 2012; Burzyn et al., 2013). Despite these examples, the functional role of ErbB/HER ligands in shaping anti-tumor immune responses *in vivo* is poorly defined though the ability of these ligands to influence the APM and MHC expression places them in a central position to do so.

As mentioned above, in the 1980s and 1990s, several groups found that treatment of cells with ErbB/HER ligands such as EGF and TGFA repress the expression/induction of MHCI and MHCII molecules (Schreiber et al., 1984; Lahat et al., 1992; Mitra and Nickoloff, 1992). More recently, we have also shown that EGF can repress the induction of MHCII molecules by IFN-γ (Pollack et al., 2011). Likewise, others have shown similar effects of ErbB/HER ligands. For example, in their studies on esophageal and gastric cancer, Mimura et al. demonstrated that MHCI was repressed by the neuregulin NRG-1-β1 (Mimura et al., 2013). Likewise, in their studies on prostate cancer, Chen et al. (2015) showed that EGF could repress MHCI expression. However, in these and other studies, not all cells respond to ErbB/HER ligands in the same manner; cells responding to one ligand may fail to respond to another, and in some cells, ErbB/HER ligands have no effect on MHC expression (Mimura et al., 2013; Vantourout et al., 2014; Chen et al., 2015). Such differences in the responses to ErbB/HER ligands are likely due to the complex nature of ErbB/HER signaling and MHC expression. For example, the cellular response to ErbB/HER ligands depends on ErbB/HER expression patterns and is likely influenced by the (activation) status of downstream enzymes (such as RAS and RAF) and other proteins that impact the response to ErbB/HER inhibition (Gazdar, 2009). Moreover, as mentioned earlier, when considering the impact of ErbB/HER ligands on MHC expression both constitutive and inducible MHCI expression warrant investigation as some effects may require cytokine treatment(s) to be manifest. **Table 5** provides a summary of studies demonstrating repression of MHC molecules by ErbB/HER ligands (in at least some contexts).

**Table 5:** Examples of ErbB/HER ligands repressing MHC expression.

ERBB/HER Ligand	MHC Repressed	Reference
EGF	MHC class I	Schreiber et al., 1984
EGF	MHC class II	Lahat et al., 1992
EGF/TGFA	MHC class II	Mitra and Nickoloff, 1992
EGF	MHC class II	Pollack et al., 2011
NRG-1-b1	MHC class I	Mimura et al., 2013
EGF	MHC class I	Chen et al., 2015

**FIGURE 2:**
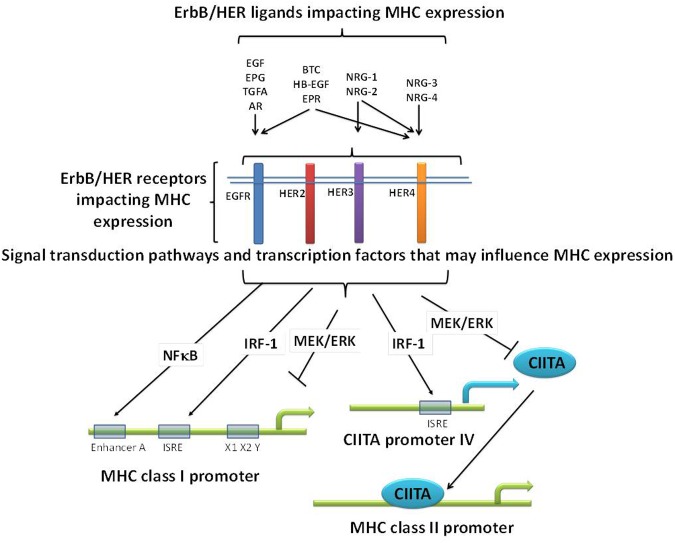
**Potential mechanisms relating ErbB/HER ligands, receptors, and MHC expression.** ErbB/HER ligands bind and activate different ErbB/HER receptors initiating distinct signal transduction pathways and cellular effects. As a result, there may be differences across ligand groups based upon the receptors and downstream signaling pathways they activate. In addition, even ErbB/HER ligands that bind the same receptors can have distinct impacts on downstream signaling and as such may have distinct effects on MHC expression. ErbB/HER ligands and receptors activate signaling pathways known to negatively (such as the MAPK pathway), and positively (such as NFκB and IRF-1), regulate MHC expression via the binding of transcription factors (TFs) to MHC promoters or the promoters of co-activators such as CIITA. As a result, in any given cellular context, the sum of these opposing effects will likely determine the impact of ErbB/HER activation and inhibition on MHC expression.

Further complicating the picture, especially as it relates to ErbB/HER-MHCI interactions, is the fact that ErbB/HER signaling activates pathways known to have opposing effects on MHC expression. For example, ErbB/HER signaling can activate NFκB, IRF-1, and Stat1, known positive regulators of MHC expression, while at the same time activate the MAPK pathway which can negatively regulate MHC expression (**Figure 2**) (Pedersen et al., 2005; Andersen et al., 2008; van den Elsen, 2011; Mimura et al., 2013; Sapkota et al., 2013; Chen et al., 2015; Shostak and Chariot, 2015). Therefore, the balance of these positive and negative regulators of MHC expression induced in response to ErbB/HER activation may ultimately dictate their impact on MHC expression. The study by Mimura et al. highlights this point. In their model, a clear hierarchy existed as EGF failed to influence MHCI expression, whereas NRG-1β1 repressed it and both the MAPK and PI3K pathways were implicated in modulating MHCI expression (Mimura et al., 2013). Thus, while many questions remain regarding the impact of ErbB/HER ligands on the APM there is growing evidence that in some contexts these ligands influence MHC expression. Moreover, based upon the expression pattern of ErbB/HER receptors present on a given tumor cell, some ligands may influence MHC expression whereas others will not. **Figure 2** illustrates some of the above concepts.

### ErbB/HER Receptors, Inhibitors, and MHC Expression

The four members of the ErbB/HER family include the EGFR (also known as ErbB1 and HER1), ErbB2 [also known as HER2 and Neu, ErbB3 (HER3), and ErbB4 (HER4)] (Roskoski, 2014). Structurally, these receptors contain an extracellular ligand-binding domain, a transmembrane domain, and a cytoplasmic domain that contains tyrosine kinase activity (Lemmon et al., 2014). There are some noteworthy exceptions to the preceding description. Namely, ErbB2/HER2/neu has no known ligand, and ErbB3/HER3 has impaired tyrosine kinase activity. ErbB/HER receptors can signal as homo- or heterodimers creating an array of combinations that have distinct signaling outputs (Roskoski, 2014). Moreover, hierarchies exist such that the ErbB2/ErbB3 heterodimer is felt to be the most potent ErbB pairing signaling combination (Pinkas-Kramarski et al., 1996; Tzahar et al., 1996; Baselga and Swain, 2009). ErbB/HER receptors are widely expressed on normal and malignant cell types (Salomon et al., 1995). Genetic knockout of ErbB/HER receptors in mice leads to alterations in numerous tissues (**Table 6**). In addition to their classical roles in regulating cellular proliferation, survival, angiogenesis, apoptosis, and the cell cycle, these receptors have recently been implicated in the control of epithelial-to-mesenchymal transition (EMT) (Chen et al., 2015). Thus, ErbB/HER family members can regulate a variety of complex processes including immune responses as outlined below.

**Table 6:** Tissues altered by genetic knockout of ErbB/HER receptors

Receptor	Tissues impacted by genetic knockout	Reference
ErbB1/HER1/EGFR	Epidermis, mammary gland, lung, pancreas, gastrointestinal tract, central and peripheral nervous system, immune system (regulatory T cells)	Miettinen et al., 1995; Sibilia and Wagner, 1995; Threadgill et al., 1995; Sibilia et al., 1998; Wiesen et al., 1999; Normanno et al., 2003b
ErbB2/HER2/NEU	Mammary gland, heart, central nervous system	Lee et al., 1995; Xie et al., 1997; Jones and Stern, 1999; Park et al., 2001; Crone et al., 2002; Leu et al., 2003
ErbB3/HER3	Heart, central and peripheral nervous system, mammary gland	Erickson et al., 1997; Troyer and Lee, 2001
ErbB4/HER4	Epidermis, mammary gland, myocardium, central and peripheral nervous system	Gassmann et al., 1995; Jones et al., 1999; Golub et al., 2004

Before reviewing the immune effects of ErbB/HER proteins, it is worth highlighting the fact that these proteins have been examined from markedly different yet valid perspectives (Seliger and Kiessling, 2013). From the cancer biology perspective, ErbB/HER proteins are canonical oncogenic enzymes that drive tumor cell proliferation/survival/angiogenesis and metastasis (Hynes and Lane, 2005). This perspective considered little the immune effects of ErbB/HER proteins yet fueled enormous efforts to inhibit ErbB/HER expression and/or activity (Mendelsohn and Baselga, 2000; Normanno et al., 2003a). Contrasting this view is the tumor immunology perspective that considers ErbB/HER proteins as bonafide tumor antigens (Zaks and Rosenberg, 1998; Nistico et al., 1999; Disis et al., 2000). From this perspective, ErbB/HER-derived peptides including those derived from activating mutations, such as the EGFRvIII variant with a mutation in the extracellular domain of the receptor, have the potential to stimulate an anti-tumor immune response innately or via vaccination (Li and Wong, 2008; Nedergaard et al., 2012; Paff et al., 2014; Schneble et al., 2014). Vaccine studies using a 14-amino acid peptide spanning the mutated extracellular domain of EGFRvIII (CDX-110) fused to adjuvant have shown durable oncogene-specific antibody and CD8 T cell responses as well as improved survival in glioblastoma multiforme patients (Sampson et al., 2008). In this case, the expression of ErbB/HER proteins on tumor cells would be required for successful anti-cancer therapy and by targeting mutations specific to cancer cells these therapies result in minimal bystander damage of healthy somatic cells expressing normal ErbB/HER proteins. Other studies incorporate both perspectives by investigating interactions between oncogenic ErbB/HER proteins and proteins central to immune responses; namely MHC molecules.

Attempts to establish links between the expression of ErbB/HER family members and MHC molecules have been the subject of study for many years (Nouri et al., 1995; Nistico et al., 1997; Lollini et al., 1998). Using a forced-expression approach, Herrmann et al. (2004) demonstrated that the expression of ErbB2/HER2 was associated with decreases in MHCI and other components of the APM. Complementing these findings using siRNA-based approaches, Choudhury et al. (2004) demonstrated that the loss of ErbB2/HER2 was associated with increases in MHCI molecules. These studies provided links between ErbB/HER proteins and MHC expression. The development of pharmacologic ErbB/HER inhibitors has provided additional evidence supporting interactions between ErbB/HER proteins and MHC molecules.

Because MHC molecules govern interactions between tumor cells and CD4 and CD8 T cells, early studies examined the effects of ErbB/HER inhibition on MHC expression. In one early study, no effect of the anti-HER monoclonal antibody (mAb) trastuzumab on MHC expression was seen though the treatment did enhance tumor lysis by MHCI-restricted cytotoxic T lymphocytes (CTLs) (Kono et al., 2004). Our group explored the impact of EGFR inhibitors on MHCI and MHCII expression and found that small molecule tyrosine kinase inhibitors (TKIs) (such as PD168393 and AG1478) as well as the EGFR blocking antibody cetuximab (alone or with the ErbB2/HER2/neu blocking antibody trastuzumab) could enhance the induction of MHCI and MCHII molecules by IFN-γ (Pollack et al., 2011). In some experimental models, the addition of either PD168393 or cetuximab alone augmented MHCI even in the absence of IFN-γ. Subsequent studies have confirmed that at least under some circumstances ErbB/HER inhibitors can enhance MHCI and/or MHCII expression. For example, erlotinib, cetuximab and the pan ErbB/HER inhibitor dacomitinib increased the induction of MHCII molecules by IFN-γ in head and neck cancer cell lines (Kumai et al., 2013, 2015). In their study on non-small cell lung cancer, Okita et al. (2015) found that gefitinib upregulated MHCI expression in some cellular contexts. Moreover, anti-ErbB2/HER2/Neu therapy increased MHCII levels *in vivo* (Mortenson et al., 2013). Further illustrating relevant functional interactions between ErbB/HER inhibition and MHC molecules was a study demonstrating that EGFRI resistance is associated with defects in MHCI expression (Garrido et al., 2014). The above studies illustrate that under some contexts, ErbB/HER proteins and inhibitors thereof can influence the expression of MHC molecules. **Table 7** lists studies reporting increases in MHCI and/or MHCII by ErbB/HER inhibition.

**Table 7:** Examples of ErbB/HER inhibitors augmenting MHC expression.

Inhibitor(s)	Type of inhibitor	Target(s)	MHC molecule increased	Reference
siRNA against ErbB2/HER2	siRNA	ErbB2/HER2	MHC class I	Choudhury et al., 2004
PD168393	Irreversible kinase inhibitor	EGFR/ErbB/1/HER1 ErbB2/HER2/neu	MHC class I and MHC class II	Blair et al., 2007; Pollack et al., 2011
AG1478	Reversible kinase inhibitor	EGFR/ErbB/1/HER1	MHC class I and MHC class II	Zhou and Brattain, 2005; Pollack et al., 2011
Cetuximab	Blocking antibody	EGFR/ErbB/1/HER1	MHC class I and MHC class II	Pollack et al., 2011
Trastuzumab	Blocking antibody	ErbB2/HER2/neu	MHC class I and MHC class II	Pollack et al., 2011
Erlotinib	Reversible kinase inhibitor	EGFR/ErbB1 ErbB2/HER2/neu	MHC class II	Schaefer et al., 2007; Kumai et al., 2013
Cetuximab	Blocking antibody	EGFR/ErbB1	MHC class II	Kumai et al., 2013
Anti-ErbB2/HER2/Neu	Blocking antibody	ErbB2/HER2/Neu	MHC class II	Mortenson et al., 2013
Dacomitinib	Irreversible kinase inhibitor	EGFR/ErbB1/HER1 ErbB/HER2/neu ErbB4/HER4	MHC class II	Kumai et al., 2015
Gefitinib	Reversible kinase inhibitor	EGFR/ErbB1/HER1 ErbB2/HER2/neu	MHC class I	Hirata et al., 2005; Okita et al., 2015

**FIGURE 3:**
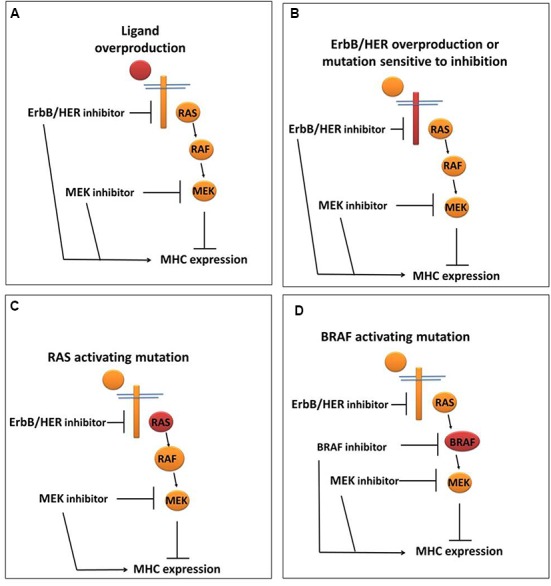
**ErbB/HER downstream signaling pathways may influence the ability of ErbB/HER and other kinase inhibitors to modulate MHC expression.** In settings where the MAPK pathway is actively repressing MHC expression, the location of the activating/oncogenic event (indicated in red) will likely influence the response to ErbB/HER and/or other kinase inhibitors. **(A)** In the setting of ErbB/HER ligand overproduction, inhibition of ErbB/HER activity (via an ErbB/HER inhibitor) and/or enzymes in the MAPK signaling pathway may increase MHC expression. **(B)** In the setting of ErbB/HER activating mutations and/or overproduction, an ErbB/HER inhibitor and/or MAPK inhibitor may increase MHC expression. **(C)** In the setting of an activating RAS mutation, the effect of an ErbB/HER inhibitor may be null/minimal because MAPK signaling is being driven by activated RAS, whereas a downstream MAPK inhibitor may increase MHC expression. **(D)** In the setting of an activating mutation in BRAF, as with an activating RAS mutation, an ErbB/HER inhibitor would not likely change MHC expression whereas a BRAF inhibitor and/or a MEK inhibitor may increase MHC expression.

While the above provide evidence that ErbB/HER activity impacts MHC expression, it is important to note that just as was the case for ErbB/HER ligands, not all cells respond to ErbB/HER inhibition with increases in MHC expression. We and others have shown that treatment of some cells with inhibitors of ErbB/HER proteins does not alter MHC expression (Pollack et al., 2011; Mimura et al., 2013; Okita et al., 2015). Thus, in some cells, ErbB/HER activity is coupled to MHC expression, whereas in other cells this is not the case. While the mechanisms underpinning these interactions are still being explored, some cells that are unresponsive to ErbB/HER inhibition respond to inhibitors of downstream enzymes particularly those inhibiting enzymes in the mitogen-activated protein kinase (MAPK) pathway as outlined below and shown in **Figure 3**.

### ErbB/HER Downstream Signaling and MHC Expression

ErbB/HER proteins are signaling hubs that can initiate signal transduction via a variety of downstream pathways that include the RAS/RAF/MAPK pathway, the phosphoinositide-3-kinase (PI3K) pathway, the phospholipase C-γ (PLC-γ) pathway and signal transducers and activators of transcription (STATS) (Pines et al., 2010). Evidence for direct links between enzymes downstream of ErbB/HER proteins, such as RAS, and the APM have existed for over twenty years (Seliger et al., 1988, 1996, 1998; Seliger and Pfizenmaier, 1989; Lohmann et al., 1996). In addition, links between RAS mutations and MHC expression have been reported in colorectal carcinoma (Atkins et al., 2004). More recently, inhibitors of the aforementioned pathways have been exploited to examine interactions between these pathways and MHC expression. Akin to the development of ErbB/HER inhibitors, the main paradigm fueling the development these inhibitors (such as those targeting BRAF^V600E^ and MEK to inhibit the MAPK pathway and those targeting the PI3K pathway) centered on their potential to block proliferative and survival signals rather than their immune effects (Rodon et al., 2013; Zhao and Adjei, 2014; Sidaway, 2015; Thorpe et al., 2015). Despite this, there is clear evidence that inhibition of enzymes downstream of ErbB/HER receptors, especially the MAPK pathway, alters MHC expression in some contexts.

Early studies using pre-clinical MEK inhibitors (such as PD98059) revealed that MEK inhibition augments MHCI and MHCII expression (as well as CD86, CD80, and CD40) on and allostimulation by growth factor-dependent DC in the presence of TNF-α (Yanagawa et al., 2002). Likewise, another group reported that PD98059 could increase MHCII expression on monocyte-derived DCs (moDCs) (Aiba et al., 2003). These studies provided early links between the MAPK pathway and MHC expression in the context of professional APCs. As mentioned earlier, similar to tumor cells, microbes such as Mtb can down-regulate MHC expression to avoid detection by the immune system. Mechanistically, the MAPK pathway has been implicated since the down regulation of the MHCII master regulator CIITA in macrophages by Mtb was mediated by MAPK signaling and reversed by the MEK inhibitor U0126 (Pennini et al., 2006). These observations support subsequent studies demonstrating that MAPK inhibitors influence MHC expression in the context of cancer.

Kono and colleagues reported that the expression of melanoma differentiation antigens (MDAs) increased in the presence of U0126 and PD98059 (Kono et al., 2006). While they found no effect on MHC expression, a subsequent study by Sers et al. (2009) demonstrated that treatment of cells with the MEK inhibitor U0126 led to increases in MHCI expression. The FDA approval and clinical use of BRAF^V600E^ and MEK inhibitors (such as vemurafenib, dabrafenib, and trametinib) have further underscored the immune effects of MAPK inhibition in the context of cancer. For example, vemurafenib induced changes in the expression of melanoma differentiation antigens (MDA), and the tumor microenvironment in patients including increases in CD8+ T cell infiltration (Boni et al., 2010; Frederick et al., 2013). Again, in these studies, no changes in MHC expression were seen, yet they underscored the potent immunologic effects induced by disruption of oncogenic MAPK signaling.

Our group looked at the ability of vemurafenib to influence the induction of MHCI and MHCII molecules by IFN-γ and IFN-γ2b. We found that in BRAF^V600E^ mutant melanoma cell lines lacking wild type BRAF the induction of MHCI and MHCII was enhanced in the presence of vemurafenib at nanomolar concentrations and that basal levels of MHCI decreased with the forced expression of BRAF^V600E^ (Sapkota et al., 2013). Further support identifying the MAPK pathway (and phospho-ERK in particular) as a dominant regulator of MHCI expression (in esophageal and gastric cancer) comes from the study by Mimura and colleagues (Mimura et al., 2013). More recently, others have reported increases in MHC expression when oncogenic MAPK signaling is disrupted especially in the context of cytokine treatment or adoptive cell therapy (Mimura et al., 2013; Hu-Lieskovan et al., 2015; Sabbatino et al., 2016; Whipple et al., 2016). Thus, inhibitors of MAPK signaling can influence the expression and induction of MHC molecules in some settings through several possible mechanisms as outlined below. **Table 8** lists examples where inhibitors of enzymes in the MAPK pathway increase MHC expression.

**Table 8:** Inhibition of enzymes in the MAPK pathway enhancing MHC expression

Inhibitor(s)	Enzyme inhibited	MHC molecule increased	Reference
PD98059	MEK1MEK2	MHC class I and MHC class II	Yanagawa et al., 2002
PD98059	MEK1MEK2	MHC class II	Aiba et al., 2003
U0126	MEK1MEK2	MHC class I	Sers et al., 2009
Vemurafenib	BRAF^V600E^	MHC class I and MHC class II	Sapkota et al., 2013
PD98059	MEK1MEK2	MHC class I	Mimura et al., 2013
Dabrafenib	BRAF^V600E^	MHC class I	Hu-Lieskovan et al., 2015
Trametinib	MEK1MEK2	MHC class I	Hu-Lieskovan et al., 2015
Vemurafenib	BRAF^V600E^	MHC class I	Sabbatino et al., 2016
Vemurafenib	BRAF^V600E^	MHC class I	Whipple et al., 2016
U0126	MEK1MEK2	MHC class I	Whipple et al., 2016

One mechanism implicated in mediating MAPK-MHCII interactions is through the activity of the MHCII co-activator (and ‘master regulator’) CIITA. CIITA is critical to the generation of immune responses as it is responsible for regulating the expression of MHC class II molecules (Choi et al., 2011). CIITA does not directly bind DNA, but instead regulates transcription by interacting with TFs and elements of the enhanceosome complex affecting chromatin remodeling and transcription initiation (Reith and Boss, 2008; Devaiah and Singer, 2013). The activity of CIITA is highly regulated through numerous post-translational modifications including ubiquitination, acetylation, and phosphorylation (Wu et al., 2009; Morgan et al., 2015). Early work demonstrated that post-translational phosphorylation of various serine residues of CIITA, were crucial to its ability to localize to the nucleus and increase MHC II expression (Greer et al., 2004). In this study, Greer and colleagues demonstrated that the loss of these phosphorylation sites was associated with an increase in the activation of endogenous MHCII genes. This finding was subsequently confirmed using dominant negative proteins and the ERK inhibitor PD98059 both of which increased MHC II induction by CIITA by attenuating ERK activity (Voong et al., 2008). Thus, while many questions remain, the MAPK pathway likely impacts MHCII expression in part via changes in CIITA post-translational modifications.

Compared to MHCII, the mechanisms through which MHCI is regulated by MAPK signaling is less well defined. Analogous to CIITA for MHCII, NLRC5/CITA has been shown to be a key regulator of MHCI expression that has been implicated in cancer (Meissner et al., 2010; Kobayashi and van den Elsen, 2012; Yoshihama et al., 2016). We have found that vemurafenib increases in NLRC5/CITA mRNA induction by IFN-γ (Sapkota et al., 2013). In addition to possible effects on NLRC5/CITA, others have identified a novel gain-of-function activity of BRAF^V600E^ that directly targets MHCI protein for degradation (Bradley et al., 2015). This study underscores the intimate links that exist between oncogenes and MHC expression.

## Incorporating Informatics to Help Understand ErbB/HER – MHC Interactions

Given the complex nature of oncogenic signaling and the regulation of immune responses, many complementary approaches need to be used in order to fully understand how ErbB/HER oncogenic signal transduction influences the expression of MHC molecules and the APM. These will likely include biochemical studies, cell-based studies, murine models, and a detailed analysis of human samples. While bioinformatics approaches are integral to all of the aforementioned models, they are particularly useful in the setting of large tumor tissue databases (Cooper et al., 2015; Li et al., 2015). Having databases containing genomic, transcriptomic, and proteomic information provides an invaluable opportunity to characterize relationships between genomic events and those relevant to immunity in the context of human cancer (Rutledge et al., 2013; Saba et al., 2015). An example relevant to MHCI expression and regulation in human cancer is outlined below.

NLRC5/CITA was known to be an important regulator of MHCI molecules, yet until recently, its role in the setting of human cancer was poorly defined. In a recently published study by Yoshihama and colleagues, our understanding of the role of NLRC5 in human cancer was greatly expanded (Yoshihama et al., 2016). The authors in this study included bioinformatics-based approaches to assess the expression pattern of NLRC5/CITA and MHCI in human cancer thereby providing a much better understanding of how NLRC5/CITA expression correlates with other immune parameters in the setting of human tumors. Moreover, these authors used this approach to determine that expression of NLRC5/CITA correlated with survival in several cancer types including melanoma, rectal cancer, bladder cancer, uterine cancer, cervical cancer and head/neck cancer. This study illustrates how genetic information from human tumor databases can be combined with bioinformatics to generate clinically relevant information. A brief description of some of these resources and approaches are reviewed in the next paragraph.

A number of public bioinformatics resources exist to investigate the relationship between immune parameters, oncogenic driver mutations and signaling network activation. The Cancer Genome Atlas^1^ is a public data resource of the National Cancer Institute that has produced comprehensive genomic, epigenomic, transcriptomic, and proteomic profiles of over 11,000 patients with 33 distinct cancer types. The data generated by TCGA is readily accessible via a number of stand-alone public resources that facilitate exploration of mutational spectra and copy number events, gene expression and their relationship to clinical outcomes including the cBioPortal^2^, the Broad Institute Tumor Portal^3^, and the Cancer Regulome Explorer^4^(Cerami et al., 2012; Gao et al., 2013; Lawrence et al., 2014). The use of these resources and others will provide important avenues to examine interactions between ErbB/HER ligands, receptors, and the APM to understand interactions between these important oncogenes and adaptive immune responses in a more comprehensive manner.

## Conclusion

In summary, functional links between ErbB/HER ligands, receptors, and MHC molecules demonstrates the intimate connections that can exist between oncogenes and genes that regulate antigen processing and presentation. The clinical use of ErbB/HER inhibitors provides a unique opportunity to define these relationships in more depth and to gain insight into how signaling pathways defined for their role in cancer influence fundamental immunologic processes. This information will enhance our ability to use targeted therapies with more insight and more rationally combine them with immune-based therapies.

## Author Contributions

AK, drafting and conception of the review; MS, critical feedback for the review; LC, drafting and conception of the review; HK, critical feedback for the review; BP, conception, drafting, and final approval of the review.

## Conflict of Interest Statement

The authors declare that the research was conducted in the absence of any commercial or financial relationships that could be construed as a potential conflict of interest.

## Footnotes

1 http://cancergenome.nih.gov

2 http://www.cbioportal.org/

3 http://www.tumorportal.org/

4 http://explorer.cancerregulome.org/
